# The impact of economic recession on the association between youth unemployment and functional somatic symptoms in adulthood: a difference-in-difference analysis from Sweden

**DOI:** 10.1186/s12889-016-2917-0

**Published:** 2016-03-05

**Authors:** Anna Brydsten, Anne Hammarström, Miguel San Sebastian

**Affiliations:** Department of Public Health and Clinical Medicine, Unit of Epidemiology and Global Health, Umeå University, SE-901 87 Umeå, Sweden

**Keywords:** Youth unemployment, Recession, National unemployment, Longitudinal analysis, Functional somatic symptoms, Difference-in-difference analysis, Northern Sweden Cohort, Sweden

## Abstract

**Background:**

The impact of macroeconomic conditions on health has been extensively explored, as well as the relationship between individual unemployment and health. There are, however, few studies taking both aspects into account and even fewer studies looking at the relationship in a life course perspective. In this study the aim was to assess the role of macroeconomic conditions, such as national unemployment level, for the long-term relationship between individual unemployment and functional somatic symptoms (FSS), by analysing data from two longitudinal cohorts representing different periods of unemployment level in Sweden.

**Methods:**

A difference-in-difference (DiD) analysis was applied, looking at the difference over time between recession and pre-recession periods for unemployed youths (age 21 to 25) on FSS in adulthood. FSS was constructed as an index of ten self-reported items of somatic ill-health. Covariates for socioeconomics, previous health status and social environment were included.

**Results:**

An association was found in the difference of adult FSS between unemployed and employed youths in the pre-recession and recession periods, remaining in the adjusted model for the pre-recession period. The DiD analysis between unemployed youths showed that men had significantly lower adult FSS during the recession compared to men in the pre-recession time.

**Conclusions:**

Adulthood FSS showed to be significantly lower among unemployed youths, in particular among men, during recession compared to pre-recession times. Since this is a fairly unexplored research field, more research is needed to explore the role of macroeconomic conditions for various health outcomes, long-term implications and gender differences.

## Background

In the 1990s, Sweden faced its worst economic and unemployment crisis since the 1930s, with dramatic changes in the labour market and the public economy. National unemployment levels rose dramatically, the labour market insurance system (disability, sickness and unemployment insurance) was slimmed down, and social inequalities increased [1–3]. One of the most alarming effects related to this recession was the high youth unemployment level. For young people at the onset of entering the labour market, it meant a higher risk of unemployment and lower occupational income, a considerable delay of independence from parents, with implications for independent living arrangements and the opportunity to start a family, as well as financial vulnerability because of the downsized social security [2, 3]. Additionally, youths as a group are more sensitive to changes in the labour market than the adult population, due to lack of experience, network and qualifications needed, with higher risks of further unemployment in adulthood [4, 5].

The relationship between unemployment and health status has been explored in a number of fields (such as sociology, economics and social epidemiology) and over an extended period of time [6–8]. Overall, unemployment has been associated with health problems, such as anxiety, depression, heart disease, hypertension, somatic ill-health and mortality, showing both short-term and long-term health implications [6, 7, 9, 10]. However, little is known about the macroeconomic impact on the association between individual unemployment and health status, particularly regarding potential long-term health implications. This is partly because of the dominance of cross-sectional studies [11].

Theories of individual level pathways suggests that recessions could have harmful short-term effects on health due to increase in stress and fear of unemployment, potential income loss and social insecurity [12]. On the other hand, a recession may reduce work hazards and risk-taking behaviours, and may increase (non-market) leisure time, leading to short-term population health improvements [13]. The few studies within the field have shown both positive and negative short-term health implications [14]. A recent Spanish study applied a difference-in-difference (DiD) approach and found a short-term effect on mental health for unemployment during the current European recession compared to unemployment during the pre-recession period [15]. An Australia study observed an increase of suicides among unemployed young men during times of recession [16]. Conversely, this pattern has not been found in the Nordic context [17]. In Sweden, unemployed during the 1990s recession showed no health impact compared to unemployment during the post-recession time [18] and in Finland suicide mortality decreased among unemployed during the recession [19]. According to a study conducted on the same cohorts as in the current study, macroeconomic conditions did not seem to be an important factor for short-term health status [20]. However, this study, like most studies within the field, focused only on current effects.

In the present study the aim was to assess the role of macroeconomic conditions, such as the national unemployment level, for the long-term relationship between individual youth unemployment and adult health status in a Swedish context, by analysing data from two longitudinal cohorts from northern Sweden. The cohorts represent different macroeconomic periods, the pre-recession and the recession, for youths in the school-to-work transition. When participants in what we henceforth call ‘the pre-recession cohort’ entered the labour market in the beginning of the 1980s, the national unemployment rate was relatively low (3.5 % at the highest level) [21]. For participants in what we label ‘the recession cohort’, the circumstances were dramatically different in the beginning of the 1990s when the unemployment levels was high (8.5 % at the highest level in 1993) [21]. For young people (age 16 to 24) this was even more dramatic, with a youth unemployment rate as high as 8.0 % during the pre-recession and 18.4 % during the recession [21].

To the best of our knowledge, this is the first study taking a life course perspective on the impact of macroeconomic conditions on the association between individual unemployment and health status.

## Method

### Participants and data collection

Prospective data have been used from two cohorts from a medium-sized industrial town in northern Sweden. The cohorts consist of all pupils who studied in the 9^th^ grade of compulsory school in 1981 (pre-recession cohort, *n* = 1083) and 1989 (recession cohort, *n* = 897). In the pre-recession cohort, data were collected at age 16, 18, 21, 30 and 42. At the last follow-up the response rate (of those still alive *n* = 1071) was 93.5 % (*n* = 1001). In the recession cohort, designed to be comparable to the pre-recession cohort, data were collected at age 21 and 39, with a response rate of 85.8 % (*n* = 686) at the last follow-up (of those still alive participating at 21, *n* = 800). The exceptionally high response rate is due to an intensive effort to contact all participants. At each follow-up, an identical questionnaire was carried out in both cohorts with around 90 questions concerning labour market, family situation and socioeconomic conditions, health and health behaviour. The questionnaires and the data collection were performed in the same way for both cohorts and merged into one dataset. A more detailed description of the cohorts, the questioners and available variables is published elsewhere [20, 22–24]. All participants provided informed consent at all follow-ups. The Regional Ethical Review Board in Umeå, Sweden, approved the data collection in this study.

### Measurements

#### Exposures

The macroeconomic condition is an unobserved variable, operationalised as the assignment to the different cohorts of low and high national unemployment levels at age 21. Individual unemployment was defined as *lack of employment, actively looking for a job and being available to the labour market*. In this study, we focus on young adults between age 21 and 25 (referred to as youths) because this age group have access to available labour market measures in Sweden but is still in a sensitive period of time where unemployment may have long-lasting health implications [25, 26]. In the recession cohort, youth unemployment was measured by register data from Statistics Sweden, while in the pre-recession cohort it was self-reported. This was due to unavailable register data from Statistics Sweden at that time [23]. In the pre-recession cohort, youth unemployment was measured at age 30 by a battery of questions about different labour market positions each semester and each summer from age 21 to age 25. If participants did not remember their previous labour market position, complementary data were collected from youth unemployment centres and youth labour market measures. The variable was coded into months in unemployment and dichotomised, in accordance to the Swedish Public Employment Service classification of long-term youth unemployment, into employed (<3 months) and unemployed (>3 months of unemployment). In the recession cohort, register data of the annual number of days in unemployment were used between age 21 and 25. All years were added and coded as months of unemployment and dichotomised as in the pre-recession cohort. Various cut-off points of unemployment have been tested (6 and 12 months) but not included in the analysis due to the low overall exposure to unemployment.

#### Health outcome

Functional somatic symptoms (FSS) at age 42 in the pre-recession cohort and age 39 in the recession cohort were measured by ten items of physical symptoms in the borderline between soma and psyche (added to an index ranging 0–20), showed to be related to internalised mental health, such as anxiety, depression and mortality [27, 28]. Self-reported symptoms during the last 12 months (headache/migraine, stomach ache, nausea, backache/hip pain/sciatica, fatigue, breathlessness, dizziness, overstrain) were asked for and answered as ‘no’, ‘yes, light’ or ‘yes, severe’. Occurrence of palpitations and sleeping difficulties were asked for and answered as ‘never’, ‘sometimes’ or ‘often/always’. Validation of the FSS measure showed good factor structure [29] (Cronbach’s alpha was 0.796 in the pre-recession cohort and 0.784 in the recession cohort).

#### Covariates

The following variables were coded equally at age 21 in both cohorts, with the exception of *parents’ occupational class* (measured at age 16 in the pre-recession cohort and age 21 in the recession cohort). All variables were merged into combined variables.

*Time spent in education* was measured by level of education, coded as ‘compulsory school’ (i.e. 9 years of education), ‘2 years upper secondary education’, ‘3–4 years upper secondary’ and ‘higher education’.

Previous health was measured by *FSS* (Cronbach’s alpha 0.70 in the pre-recession cohort and 0.74 in the recession cohort), and health behaviours as *smoking* (‘yes’ and ‘no’) [29].

Participants’ agency within the labour market was measured by two variables: *if they are doing what they want to do* (‘yes’ or ‘no’) and *outlook on the future* for the next 6 months (‘education’, ‘work’ or ‘unemployed, parental leave or other’).

Another dimension of young people’s transition into adulthood is experiences of independence from parents, which was measured by three variables: *living arrangements* (‘parents’ or ‘alone, spouse or friends’), *income* (‘own income’, ‘student loans’, ‘parents or partners income’ or ‘social benefits’) and *cash margin* (able to get hold of 5000 SEK in the pre-recession cohort and 10,000 SEK in the recession cohort in 1 week, ‘no’, ‘yes, own assets’, ‘yes, loan’ or ‘yes, otherwise’).

*Parents’ occupational class* was based on participants’ own reporting of their parents’ occupation and classified into two groups: white-collar workers and entrepreneurs and blue-collar workers including manual workers. The variable was coded as ‘both parents white-collar workers’, ‘one parent blue-collar worker’ and ‘both parents blue-collar workers’.

*Parents’ unemployment*, *parents’ health* and *unemployment in adulthood* (age 30 to 35, conducted on register data in both cohorts) were not included in the analysis due to the low prevalence and sample size.

### Analyses

Between-cohort differences regarding all study variables were assessed by independent sample t-tests and Pearson chi-squared statistics. Thereafter we applied a difference-in-difference (DiD) approach [30]. DiD is a quasi-experimental causal inference technique adaptable for estimating effect across time without randomly assigned group comparisons [31]. It is a research design based on controlling for confounding variables where time is the main difference. DiD is commonly used to evaluating the effect before and after a certain policy that do not affect everybody at the same time and in the same way [32]. In this study we applied a modified DiD approach with four different groups instead of two, an approach applied in other studies [15]. Four different groups (2 pairs of unemployed and employed) were exposed to different macroeconomic periods (pre-recession and recession) [31, 32].

A linear regression DiD equation model was used:

$$ {Y}_{idt}=\alpha +\delta Unem{p}_{it}+\lambda t+\gamma \left( Unem{p}_{it}\ast t\right)+{X}_{it}^{\prime }{\beta}_t+{\varepsilon}_{idt} $$

The main components were FSS in adulthood (represented by Y), the labour market position (δ, where δ = 0 employed and δ = 1 unemployed), the macroeconomic periods (λ, with *t* = 0 pre-recession and *t* = 1 recession and the interaction term (γ), capturing the impact of the macroeconomic periods on the relationship between unemployment status and health later in life. It also includes potential confounding covariates and an error term.

The empirical strategy is two-parted. First, an analysis was conducted separately for the pre-recession and the recession periods with two models: a crude model and an adjusted model with covariates Then a DiD analysis was carried out to assess the impact of labour market position during the pre-recession compared to the recession period for the adult FSS [30]. Difference in adulthood FSS for unemployed was attributed to the change of macroeconomic period [31, 32]. A significant test was conducted with a bootstrapping of 1000 repetitions to calculate the sampling distribution of means and the standard error for the different differences [30]. A DiD Kernel Propensity score matching was also applied as a sensitivity analysis obtaining similar results. Analyses were conducted in the total sample and separately for women and men. All analyses were performed in SPSS 22 and Stata 13.

## Results

The prevalence of unemployment differed between the cohorts (Table 1). In the pre-recession cohort, 16.1 % had at least a 3-month spell of unemployment compared to 37.3 % in the recession cohort (*p* <0.01). Cohort similarities were found in adulthood FSS (4.24 compared to 3.94, *p* = 0.07) but varied in youth (2.82 in the pre-recession period compared to 3.52 in the recession period, *p* <0.01). The descriptive statistics of the covariates showed significant differences in all variables except for gender and smoking between the cohorts (Table 1).

**Table 1:** Descriptive statistics for all study variables in the pre-recession and recession

Variables	Pre-recession		Recession		*p* ^*^
Unemployment (21 to 25 years old), (*n*=) %					
No unemployment	840	83.9	430	62.7	<0.01^b^
Unemployment	161	16.1	256	37.3	
Functional somatic symptoms, Mean (SD)					
Adulthood	986	4.24 (3.31)	671	3.94 (3.27)	0.07 ^a^
Youth	966	2.82 (2.51)	662	3.52 (2.98)	<0.01^b^
Gender,(*n*=) %					
Women	482	48.1	340	49.6	0.57^b^
Men	519	51.9	346	50.4	
Doing what they want, (*n*=) %					
Yes	417	42.1	248	36.2	0.02^b^
No	573	57.9	438	63.8	
Parents’ occupational class, (*n*=) %					
Both parents white-collar workers	300	30.0	298	43.8	<0.01^b^
One parent blue-collar worker	335	33.5	263	38.6	
Both parents blue-collar workers	366	36.5	120	17.6	
Smoking, (*n*=) %					
No	627	63.1	448	66.1	0.21^b^
Yes	367	36.9	230	33.9	
Living arrangement, (*n*=) %					
Parents	349	35.1	267	40.0	0.05^b^
Alone, spouse or friends	645	64.9	402	60.0	
Time spent in education, (*n*=) %					
Compulsory school	127	12.8	104	15.2	<0.01^b^
2 years’ secondary education	487	48.9	182	26.5	
3–4 years’ secondary education	264	26.5	213	31.05	
Higher education	118	11.9	187	27.3	
Income, (*n*=) %					
Own income	915	91.9	212	31.0	<0.01^b^
Student loans	20	2.0	174	25.4	
Parents or partners income	59	5.9	64	9.4	
Social benefits	2	0.2	234	34.2	
Cash margin, (*n*=) %					
No	276	28.6	213	31.4	<0.01^b^
Yes, own assets	477	49.4	320	47.2	
Yes, loan	186	19.6	90	13.3	
Yes, otherwise	27	2.8	55	8.1	
Outlook on the future, (*n*=) %					
Education	267	26.8	281	41.0	<0.01^b^
Work	575	57.7	218	31.8	
Unemployment, parental leave or other	154	15.5	186	27.2	

*p-value of the difference between the pre-recession cohort and the recession cohort

^a^T-test ^b^Chi^2^

The DiD analysis (Table 2) shows the estimated FSS in adulthood across the macroeconomic conditions. First between unemployed and employed youths within pre-recession and within the recession, and then the difference-in-difference between youth unemployment during the pre-recession and youth unemployment during recession.

**Table 2:** Estimated impact of macroeconomic conditions on the association between youth occupational status and FSS in adulthood (β, 95 % Confidence interval)

	Pre-recession						Recession						DiD between recession and pre-recession	
	Crude model			Full model			Crude model			Full model			Crude model	Full model
	Unemp	Emp	Diff^a^	Unemp	Emp	Diff^a^	Unemp	Emp	Diff^a^	Unemp	Emp	Diff^a^	DiD^b^	DiD^b^
Total sample (*n*=)	159	827		152	762		252	419		230	379		1657	1523
FSS	5.62	3.97	1.65***	5.33	4.06	1.27***	4.42	3.65	0.77***	3.74	3.45	0.30	−0.88**	−0.98**
Standard error	0.26	0.11		0.71	0.75		0.21	0.16		0.80	0.73			
Men (*n*=)	84	426		81	391		129	208		120	188		847	780
FSS	5.61	3.38	2.23***	4.65	2.88	1.78***	3.81	3.04	0.78**	3.19	2.79	0.41	−1.45**	−1.37**
Standard error	0.31	0.14		0.80	0.64		0.25	0.20		0.72	0.64			
Women (*n*=)	75	401		71	371		123	211		110	191		810	743
FSS	5.64	4.60	1.04**	3.85	3.15	0.69	5.06	4.26	0.80**	2.04	1.92	0.11	−0.24	−0.58
Standard error	0.41	0.18		1.12	1.12		0.32	0.24		1.12	1.14			

*** *p* < 0.01; ** *p* < 0.05

Full model adjusted for education, parents’ occupational class, smoking, FSS, living arrangement, income, doing what they want, outlook on the future and low cash margin

^a^Difference in adulthood FSS between unemployed and employed youths within the pre-recession and the recession

^b^Difference-in-difference in adulthood FSS between unemployed and employed youths in the recession and in the pre-recession

The first column of the table presents the estimated FSS in the pre-recession. The crude model showed an average significant difference between unemployed and employed in adult FSS for the total sample, women and men. The association remained for the total sample and for men in the adjusted model (1.24 in total sample and 1.73 for men, *p* <0.01) but not for women. In the second column, the estimated FSS is presented for the recession period. The crude model showed a small but significant difference between unemployed and employed on adult FSS for total sample, women and men. However, no significant difference remained in the adjusted model. The last column presents the DiD between unemployed in the pre-recession and unemployed in the recession calculating the average difference, first for the crude model and then for the adjusted model. The DiD analysis showed a statistically significant negative effect of the recession period on the relationship between youth unemployment and FSS (full model: −0.98 in the total sample and −1.37 for men, *p* <0.05), implying a lower risk of adulthood FSS during the recession compared to hard economic times. This was found in both models for the total sample and for men, but not for women.

## Discussion

This study examined the impact of macroeconomic conditions on the association between youth unemployment and FSS later in life. The findings suggest that youth unemployment during the pre-recession time had greater negative influence on the long-term health than youth unemployment during the recession. This association remained for men and the total sample after accounting for previous health status and several social and economic circumstances, but not for women.

This is, as far as we know, one of the first studies investigating this issue with a life course perspective. However, several cross-sectional studies have suggested a general harmful health effect in the labour force due to economic recession [15, 16, 33]. In particular, a recent Spanish study with similar analytical approach showed that unemployed had significant worse health status during the current economic recession compared to the pre-recession time [15]. In contrast to our results, these findings may reflect the contextual differences between Sweden and other western countries, such as in unemployment, social policy measures and health status. One profound difference is the level of national unemployment levels reported. In the Spanish study the highest level of national unemployment was 27.2 % compared to 18.4 % reported in ours.

This study shows that young people with unemployment during the recession have better health later in life, compared to unemployed youths in the pre-recession time. This pattern may be partly explained by the substantial increase of higher education during the 1990s compared to the 1980s [34]. The Swedish educational system promotes higher education for all citizens by enabling free education and a well-developed student loan system via the Swedish state. During the 1990s the educational system developed even further by doubling the number of places in higher educational degrees [34]. A comparison of the cohorts showed that young people in the recession cohort were more dependent on social benefits and also had less access to unemployment measures [3], but they also spent more time in education and strove to do so in the future, compared to the pre-recession cohort. It may be that young people in the recession cohort chose higher educational studies as a consequence of the unemployment rate at that time and the encouragement from the Swedish state. The implication could be that, compared to the pre-recession cohort, they came to be well-educated in adulthood, adaptable to the Swedish labour market, had high salary jobs with better work conditions and access to more social and economic buffers. This could be viewed as a pathway of accumulated health promotion factors, even if it may be caused by non-beneficial conditions. Another possible explanation for our findings could be the normalisation of the risk of unemployment during the recession compared to the experiences during the pre-recession time. This could be viewed in terms of less health selection into unemployment, less isolation and less social and material stigma related to unemployment, functioning as protecting factors of ill-health related to unemployment.

In this study we did not find any significant difference between pre-recession and recession among women, except in the crude DiD model. The relationship was mainly confounded by FSS at age 21, showing that the health status in youth is an important predictor of health later in life. In the recession cohort, income and education at age 21 were confounding factors among women and men, but not to the extent of eliminating the statistical association among men. This pattern of significant associations among men but not among women has been observed in previous studies [9, 35]. Studies have shown that people in Sweden are affected by unemployment in the same way regardless of gender [36], due to women’s high labour market participation and the social democratic welfare system in place. An interpretation might be that the unemployment situation by gender is channelled through different health outcomes, but more research is needed to further explore this gender difference.

### Limitations

There are a number of limitations in this study. First, the difference in health between the cohorts may be due to cohort selection. As a sensitivity analysis, we performed a DiD kernel propensity score matching in order to reduce some of these biases by including all covariates as matching variables. The analysis showed similar results supporting the DiD findings. Nevertheless, bias due to unavailable covariates may still occur. However, with only 8 years between the cohorts and no profound changes in the Swedish society or in the Swedish labour market during the time of exposure of youth unemployment, we can assume the difference found in this study may be due to the recession. Second, the pre-recession cohort has shown to be comparable to the Swedish population with regard to demographic and socioeconomic factors as well as illness and health behaviour [22], and the recession cohort appears to be comparable as well. But since the cohorts are geographically, socially and culturally located in a mid-sized town in northern Sweden, they may be more homogeneous than the Swedish population in general. Third, the different measures of unemployment may be problematic for the comparability. However, descriptive analysis of unemployment spells (self-reported and register data) in adulthood were approximately the same in the pre-recession cohort. Giving some indications of non-bias in the reporting of unemployment. Finally, the level of unemployment, which was considered as a proxy for macroeconomic conditions, is commonly used and reflects the economic and labour market conditions in a country [37]. However, because of the unobserved state of the exposure, other macroeconomic differences may be interrelating factors, such as access to different labour market measures, and the inference made should be taken cautiously.

## Conclusion

This study contributes to a fairly unexplored research field, showing an impact of macroeconomic conditions on the long-term association between youth unemployment and health in adulthood. Adulthood FSS showed to be significantly lower for unemployed youths during the recession compared to pre-recession times, particularly for men. There is however a need to further explore the role of macroeconomic conditions for various health outcomes, long-term unemployment spells and gender differences.
